# Brillouin scattering self-cancellation

**DOI:** 10.1038/ncomms11759

**Published:** 2016-06-10

**Authors:** O. Florez, P. F. Jarschel, Y. A. V. Espinel, C. M. B. Cordeiro, T. P. Mayer Alegre, G. S. Wiederhecker, P. Dainese

**Affiliations:** 1 Gleb Wataghin Physics Institute, University of Campinas, Campinas 13083-859, Brazil

## Abstract

The interaction between light and acoustic phonons is strongly modified in sub-wavelength confinement, and has led to the demonstration and control of Brillouin scattering in photonic structures such as nano-scale optical waveguides and cavities. Besides the small optical mode volume, two physical mechanisms come into play simultaneously: a volume effect caused by the strain-induced refractive index perturbation (known as photo-elasticity), and a surface effect caused by the shift of the optical boundaries due to mechanical vibrations. As a result, proper material and structure engineering allows one to control each contribution individually. Here, we experimentally demonstrate the perfect cancellation of Brillouin scattering arising from Rayleigh acoustic waves by engineering a silica nanowire with exactly opposing photo-elastic and moving-boundary effects. This demonstration provides clear experimental evidence that the interplay between the two mechanisms is a promising tool to precisely control the photon–phonon interaction, enhancing or suppressing it.

## Abstract (web summary)

The interaction between light and acoustic phonons is radically modified in sub-wavelength photonic structures. Here, the authors engineer the optical and acoustic modes to perfectly cancel the two interaction mechanisms—the photo-elastic and moving-boundary effects—which leads to Brillouin self-cancellation.

## Graphical abstract

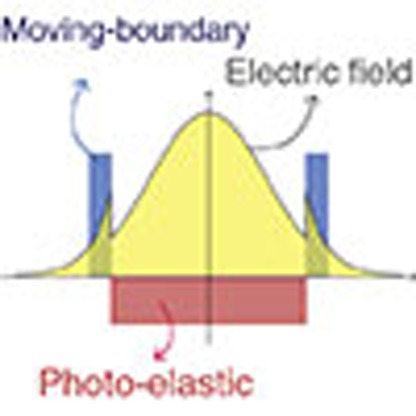

Brillouin scattering arises from the interaction between an electromagnetic wave and an acoustic wave. An incident photon at frequency ω is scattered into an up- or down-shifted photon with frequencies ω±Ω, due to the absorption or creation of a phonon with frequency Ω, respectively. The ability to strongly confine both optical and acoustic modes in photonic structures has opened new opportunities to control their interaction^1^,^2^, and several new or enhanced applications have been explored, such as in photonic signal processing^3^,^4^,^5^,^6^,^7^, demonstration and tailoring of slow-light devices^8^,^9^,^10^,^11^, actuation and control of photonic structures using optical forces^12^,^13^,^14^, observation of Raman-like scattering^15^,^16^, on-chip Brillouin scattering^17^,^18^,^19^,^20^, generation of frequency combs^21^,^22^, controlling the electrostriction properties of a medium using metamaterials^23^ and new sensing applications^24^. This interaction has also been a platform for fundamental physics experiments such as coherent phonon generation^25^, cooling mechanical modes to ground state^26^,^27^, generation of squeezed-light states^28^ and synchronization of micromechanical oscillators^29^.

Traditionally, the most common interaction mechanism between the optical and acoustic fields in low-contrast waveguides is the photo-elastic effect (pe-effect), a well-known volume effect in which the acoustic strain fields perturb the material's refractive index^30^. For example, this effect dominates Brillouin scattering in standard optical fibres^31^. However, as the surface-to-volume ratio becomes larger and the index contrast higher, such as in nano-optical waveguides and cavities, the optical field experiences an additional surface effect due to the vibrating boundary^32^,^33^,^34^,^35^, referred to as moving-boundary effect (or mb-effect). With these two effects simultaneously perturbing the dielectric constant, precise engineering of the optical and acoustic modes in a certain structure can be used to control their interaction, that is, enhancing or suppressing it. In (ref. 34), an analogous argument in terms of optical forces (electrostriction and radiation pressure) has been theoretically explored for a variety of structures and materials, demonstrating the richness of such interplay between the two mechanisms.

In the following, we demonstrate experimentally that we can achieve exact cancellation of backward Brillouin scattering by simply varying the wire diameter. It is interesting to note that physically all conditions favour strong photon–phonon interaction, phase-matching condition is satisfied, both optical and acoustic fields are strongly confined and highly overlapping spatially, and, most importantly, individually each interaction mechanism, pe- or mb-effect, is strong. However, due to a precise control of the acoustic and optical mode profiles, the surface vibrations produce a dielectric perturbation that cancels out exactly the perturbation caused by internal body strain vibrations. We refer to this effect as the Brillouin scattering ‘self-cancellation' (BSC), since the cancellation arises from the same acoustic mode that creates each effect individually. Experimentally demonstrating the BSC effect validates our fundamental understanding of the photon–phonon interaction in sub-wavelength confinement regime, opening up the possibility to selectively suppress or further enhance the interaction by exploring both effects simultaneously.

## Results

### BSC framework

**Figure 1:**
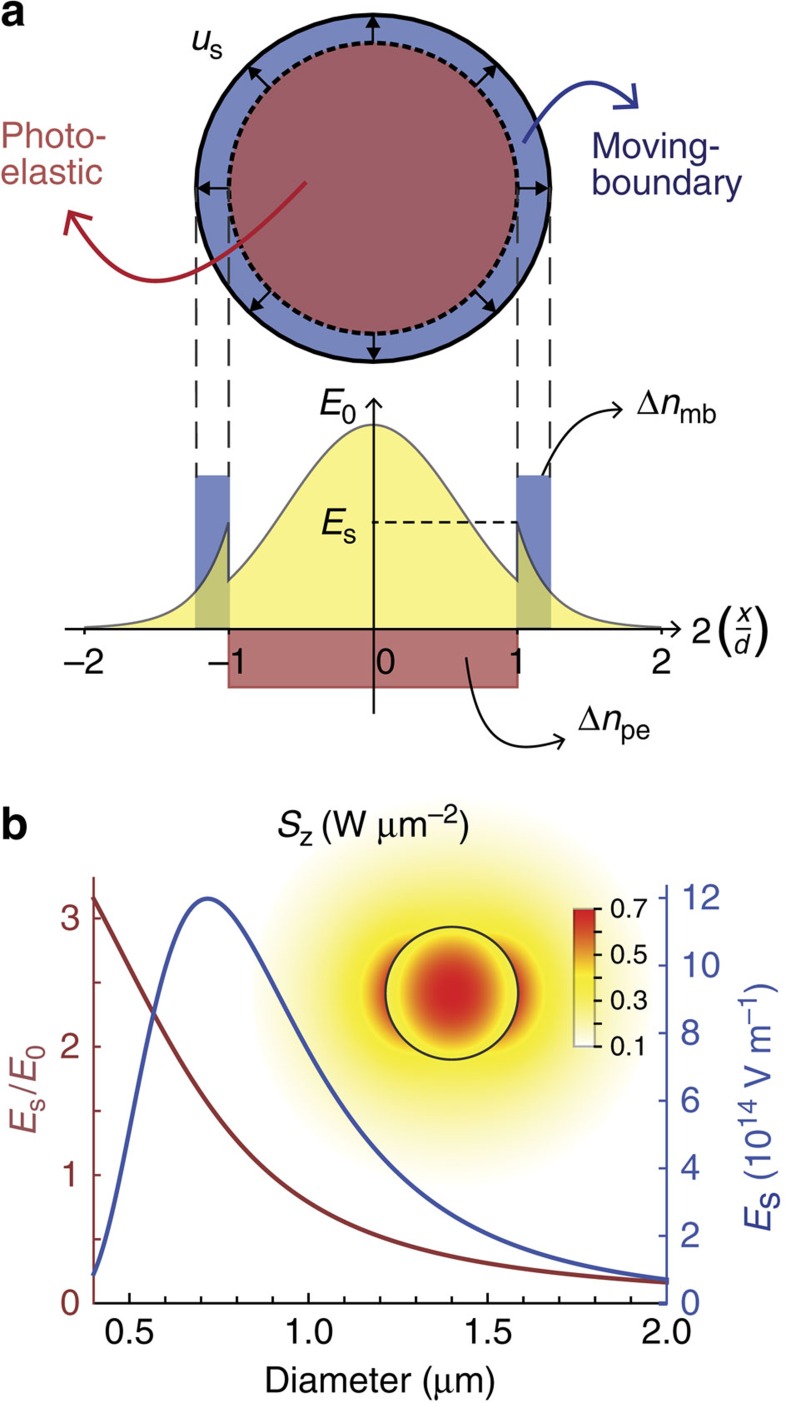
Illustration of the photo-elastic and moving-boundary effects. (**a**) Refractive index perturbation Δ*n*_pe_ due to the pe-effect (red) and Δ*n*_mb_ due to the mb-effect (blue) in a nanowire under a linear radial expansion, overlaid with the electric field profile (*d*: wire diameter, *x*: horizontal coordinate and *u*_s_: surface displacement); (**b**) surface field amplitude *E*_s_ as a function of wire diameter (blue: absolute value for 1 W optical power; red: amplitude relative to the centre field *E*_0_). Inset: *z*-component of the Poynting vector for 0.55 μm diameter.

The schematic in Fig. 1a illustrates the interplay between these two mechanisms in the case of a cylindrical-wire waveguide under a purely radial acoustic expansion. We can simply characterize the strength of the mb- and pe-effects by evaluating their index perturbation ‘*deptḣarea*' product. As the wire boundary expands out by a small surface acoustic displacement *u*_s_, the refractive index in a small region near the surface abruptly ‘increases' by Δ*n*_mb_=*n*_glass_−*n*_air_=0.45 (where *n*_glass_=1.45 and *n*_air_=1.0 are the refractive indices of the silica wire and air cladding, respectively). Therefore the strength of the mb-effect is: *η*_mb_=Δ*n*_mb_*A*_mb_=(*n*_glass_−*n*_air_)*πdu*_s_, where *A*_mb_=*πdu*_s_ is the area in which the mb-effect effect takes place (here *d* is the wire diameter). Although Δ*n*_mb_ is a relatively large perturbation, it affects only a small area (for thermally excited acoustic waves in the GHz frequency range, the boundary displacement is on the order of 10^−16^ m). Clearly, larger index contrast leads to stronger moving boundary effect.

The pe-effect, on the other hand, induces a perturbation throughout the entire wire cross-section, and so *A*_pe_=*πd*^2^/4. Despite its tensorial nature, the order of magnitude of the pe-effect can be estimated by considering only the radial strain component *S*_*rr*_ (any other deformation in the longitudinal or azimuthal direction is ignored here; however, a rigorous analysis is presented further in this article). Assuming that the radial acoustic displacement increases linearly with *r*, as *u*_*r*_=*u*_s_2*r*/*d* (with *r* being the radius coordinate), the strain becomes simply *S*_*rr*_=∂_*r*_*u*_*r*_=2*u*_s_/*d*. This then results in a index change of 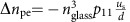 (ref. 30). In contrast to the mb-effect, a radial expansion leads to a ‘reduction' in the refractive index (this is true since silica glass has a positive photo-elastic coefficient *p*_11_=0.121 (ref. 36)). The strength of the pe-effect is then 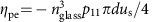. With that, we estimate that the ratio 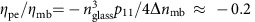. This order of magnitude estimate indicates that both effects are indeed comparable in silica nanowires and, moreover, can have opposite signs.

Obviously, this simple analysis overlooks the crucial role of the optical fields. Not only the field strength of both incident and scattered waves must be non-negligible in the region where the index perturbation occurs, but also its spatial profile plays as a weighing function for each perturbation in a first-order spatial average. Figure 1b shows the evolution of the field strength on the surface as a function of wire diameter for an optical wavelength of 1.55 μm (the inset shows the intensity profile for a 0.55 μm diameter, clearly distributed over an area larger than the wire itself). The surface field increases as the diameter is reduced, reaches a maximum and then decreases for very small diameter. This defines a region where the mb-effect might become relevant in the photon–phonon interaction, somewhere between 0.5 and 2.0 μm in diameter. This behaviour allows us to scan the nanowire diameter until we find a point in which the optical field provides just the right weighing, so that the elasto-optic and the mb-effects cancel out exactly.

### Rigorous theoretical analysis

The exact contribution from each mechanism can be calculated using standard perturbation theory (developed in detail in the Supplementary Note 1). The strength of the photon–phonon coupling through pe-effect is expressed mathematically as^36^:


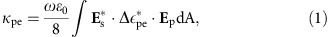


where 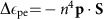 is the relative permittivity perturbation caused by the acoustic strain tensor (**S**), *n* and **p** are the material's refractive index and photo-elastic tensor, while **E**_p_ and **E**_s_ are the power-normalized electric field profiles for the pump and scattered waves. Physically, the overlap integral simply represents a spatial average of dielectric perturbation weighted by the optical fields' profiles. As discussed before, the same acoustic wave that causes the pe-effect, also causes a displacement on the boundaries that define the waveguide structure (in our case the wire boundary), and again using first-order perturbation, the moving-boundary coupling coefficient is given by^33^:


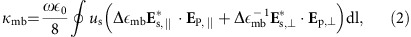


where the perturbation coefficients are 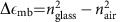 and 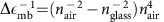. In equation 2, the normal component of the electric field is evaluated in the outer region (air cladding) to correctly take into account the field discontinuity (this is equivalent to the definition given in (ref. 33) using the continuous normal displacement field). Physically, this line integral represents an average of the dielectric perturbation in an infinitesimal area along the waveguide perimeter, weighted by the optical fields. To first order, the overall interaction strength is determined by sum of the coupling coefficients *κ*=*κ*_pe_+*κ*_mb_ and the effect of BSC is achieved when *κ*_mb_=−*κ*_pe_.

**Figure 2:**
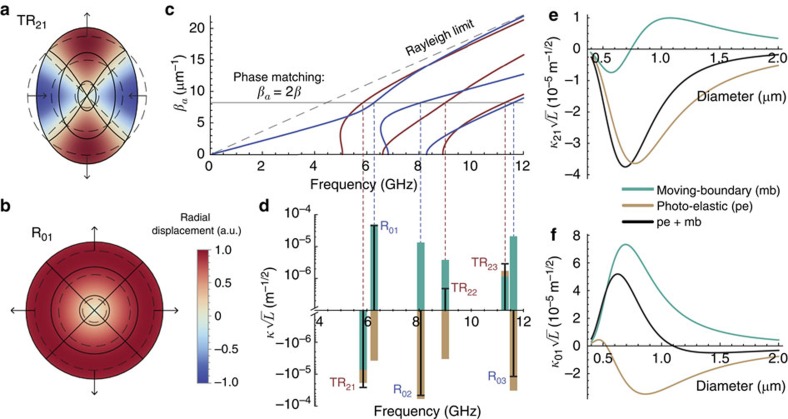
Acoustic modes and perturbation strength. Nanowire cross-section under deformation due to the two fundamental Rayleigh acoustic waves: axially asymmetric torsional-radial *TR*_21_ mode in **a** and axially symmetric radial *R*_01_ mode in **b**. Solid and dashed lines represent the deformed and un-deformed parametric lines, respectively, and the colour profile represents the normalized radial acoustic-displacement amplitude; (**c**) dispersion relation for both families (*R*_0*m*_ in blue and *TR*_2*m*_ in red) for a nanowire with 0.55 μm diameter. The horizontal continuous line represents the phase-matching condition for backward Brillouin scattering; (**d**) coupling coefficients calculated for each phase-matched acoustic mode also for a nanowire with 0.55 μm diameter (green: moving-boundary contribution; orange: photo-elastic contribution; and black: total *pe*+*mb*); (**e**,**f**) coupling coefficients as a function of the nanowire diameter. The coupling coefficients were calculated for acoustic modes normalized to thermal energy (at 300 K), and the factor *L*^1/2^ scales the normalization to any waveguide length.

To evaluate the coupling coefficients in equations 1 and 2, one must obtain the acoustic mode profiles. Each mode creates a different perturbation profile, both on the surface and throughout the wire cross-section. The acoustic modes in a cylindrical rod geometry can be categorized in symmetry-based modal families^37^, and can be calculated analytically. The fundamental optical mode interacts most efficiently with two acoustic mode families: axially symmetric radial (*R*_0*m*_) modes and axially asymmetric torsional-radial (*TR*_2*m*_) modes^38^,^39^,^40^. Such nomenclature is used here for all acoustic propagation constants, not only at the cut-off point^39^. The axially symmetric family *R*_0*m*_ exhibits only radial and axial acoustic displacements, while the *TR*_2*m*_ exhibits all three displacement components, that is, radial, azimuthal and axial. Figure 2a,b shows the wire cross-section under the deformation caused by the fundamental modes, *TR*_21_ and *R*_01_, respectively. The acoustic dispersion relation calculated for a silica wire with 0.55 μm diameter is shown in Fig. 2c. The horizontal line represents the phase-matching condition for backward Brillouin scattering *β*_*a*_=2*β* (where, *β*_*a*_ and *β* are the acoustic and optical propagation constants, respectively), and the crossing points with the dispersion curves determine the frequencies of the acoustic modes involved in the interaction. Due to such small wire diameter (from 0.5 to 2.0 μm), there are only a few acoustic modes per family, which leads to multi-peaked Brillouin spectrum^2^.

The fundamental modes in each families, *R*_01_ and *TR*_21_, are of particular interest because they have the largest acoustic displacement near the surface (thus enhancing the mb contribution). In the high-frequency limit—when the acoustic frequency is much larger than the mode cut-off frequency—the phase velocity of these two modes (*R*_01_ and *TR*_21_) approaches a certain limit, so-called ‘Rayleigh speed' (and for that these modes are referred to as Rayleigh waves)^38^,^39^. Brillouin scattering due to such Rayleigh waves has been observed recently^41^. In this limit, the Rayleigh speed is lower than the bulk longitudinal or transverse speeds, which in turn means that the transverse wavevector becomes imaginary, and the acoustic field profile decays exponentially from the surface inwards^38^. In other words, in the limit of high frequency, these modes are pure surface waves. Even below the Rayleigh limit, a large surface displacement is expected, as can be seen from the profile in Fig. 2a,b. The calculated coupling coefficients for this 0.55 μm wire diameter are shown in Fig. 2d, in which the individual contribution from each effect is shown separately for modes up to 12 GHz. Clearly, the shifting-boundary effect can not be neglected and, in fact, it dominates for some modes. Specifically, Fig. 2e,f show the calculated mb- and pe-coupling coefficients as a function of the wire diameter for the two Rayleigh waves (*R*_01_ and *TR*_21_). The pe-effect dominates the interaction for the *TR*_21_ mode throughout the entire diameter range explored here. There is no diameter in which mb-effect compensates the pe-effect, and therefore we do not expect to observe the self-cancellation effect for *TR*_21_. This can be easily understood based on the azimuthal cos2*φ* dependence, which means that the wire surface is deformed to a somewhat elliptical shape. The sign of the dielectric perturbation due to surface shift follows the radial displacement sign along the wire perimeter (that is, positive in the regions the wire expands out and negative where it is contracted). As a result, the average mb-perturbation (that is, the line integral in *κ*_mb_) is reduced. For the *R*_01_ mode, on the other hand, the mb- and pe-effects are of the same magnitude and have opposite signs, as our simple analysis had indicated. Clearly, at a diameter=1.1 μm, they exactly cancel out and no backward Brillouin scattering should be observed for this mode.

### Experimental results

**Figure 3:**
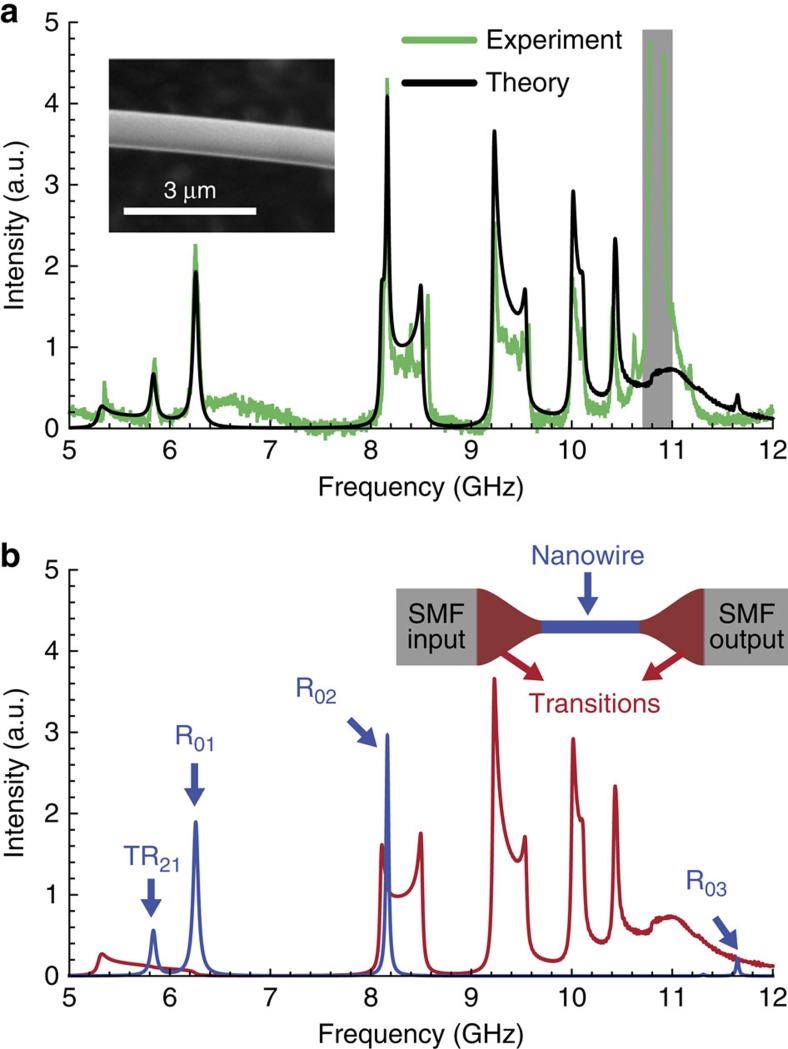
The Brillouin spectrum. (**a**) Experimental (green) and theoretical (black) Brillouin backscattering spectra for a sample with a diameter of 0.55 μm. The shaded region represents the scattering peak due to the single-mode fibre (SMF) pigtails; (**b**) contributions to the total spectrum arising from the transition regions (red) and from the actual central nanowire (blue). The theoretical spectrum in **a** is the sum of these contributions. Insets: scanning electron microscope image of a nanowire and schematic of the sample structure, indicating the SMF input/output pigtails, the transition regions and the central nanowire.

Samples of silica nanowires were fabricated by heating and stretching standard telecommunications fibre^42^. A geometric schematic is shown in the inset of Fig. 3b. Two transition regions connect the actual nanowire (central region) to single-mode fibres at both input and output ends. All nanowires are ∼8 cm long and have transition regions of ∼5 cm on each side. We report results on samples with diameter ranging from 0.51 to 1.34 μm. The precise wire diameter was determined using a non-destructive experimental technique based on forward Brillouin scattering. Both experimental setups for forward and backward Brillouin scattering characterization are described briefly in the ‘Methods' section, and additional details are given in the the Supplementary Note 2. A schematic of the experimental setup used to detect forward Brillouin scattering and an example of the detected spectrum (for a wire with 0.91 μm) are shown in Supplementary Figs 1 and 2, respectively. The setup used to detect backward Brillouin scattering is shown in Supplementary Fig. 3.

The backward Brillouin scattering spectrum obtained for a sample with 0.55 μm diameter is shown in Fig. 3a, along with the theoretical spectrum calculated for the same nanowire diameter (the theoretical linewidth were obtained directly from the experimental spectrum). The first point to highlight is that several peaks in the experimental spectrum do not correspond to the frequencies of acoustic modes calculated for a wire with the same diameter (which were shown in Fig. 2c). These measured additional peaks can be explained by considering the single-mode fibre pigtail and the transition regions. The single-mode fibre at the input gives origin to the peak at 10.8–10.9 GHz (shaded in grey as this is not the focus of this paper). In Fig. 3b, we show the calculated contributions from the nanowire with uniform diameter (in blue) and from the transition region with varying diameter along its length (in red)^42^. The transition region leads to broad scattering bands, which arise because the phase-matching frequency sweeps the acoustic dispersion curve as the diameter varies. The theoretical curve in Fig. 3a is the sum of these two curves from Fig. 3b. The theoretical spectrum explains the one observed experimentally with remarkable agreement, and no fitting parameter was required (except for a 1% adjustment on the bulk acoustic velocities to match the calculated and measured frequencies).

**Figure 4:**
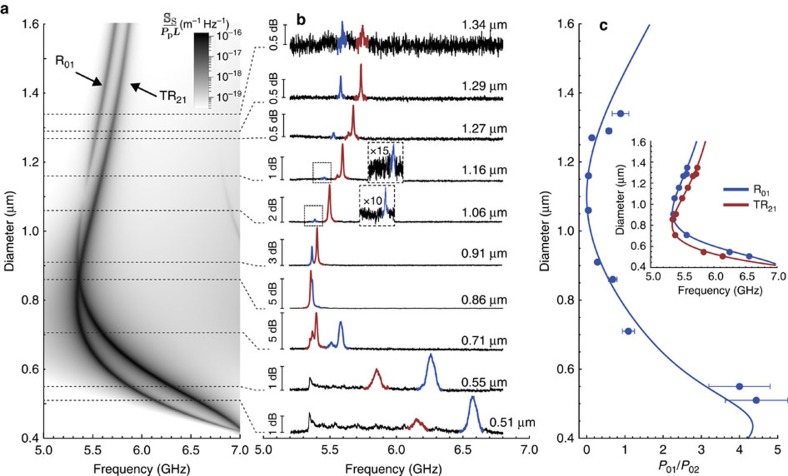
Observation of the BSC effect. (**a**) Theoretical evolution of the Brillouin backscattering spectrum as a function of the nanowire diameter for both Rayleigh waves *R*_01_ and *TR*_21_. The colour scale represents the scattered power spectral density (normalized by the pump power and wire length); (**b**) series of experimental spectra for nanowires with different diameters (as indicated in each spectrum). The peaks due to the *R*_01_ or the *TR*_21_ are coloured in blue and red, respectively; (**c**) ratio between the *R*_01_ and the *TR*_21_ total scattered power for all samples, and the theoretical result (solid curve) given by 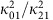. The error bars were calculated taking into account the uncertainties on the detected electrical power, on the nanowire length and on the input pump power. Inset: measured central frequencies for both *R*_01_ and *TR*_21_ (dotted blue and red points) compared with the expected theoretical frequencies (solid curves) as a function of diameter.

To demonstrate BSC in our silica nanowires, we show in Fig. 4a the calculated Brillouin spectrum evolution as a function of the nanowire diameter for the two fundamental acoustic modes (*R*_01_ and *TR*_21_). Slightly above their accidental frequency crossing (at *d*=0.9 μm), the BSC effect of the *R*_01_ mode is predicted for a roughly 50 nm diameter range around 1.1 μm. This behaviour is precisely confirmed in the series of measured spectra, shown in Fig. 4b. Each spectrum was obtained for a nanowire with a distinct diameter, as indicated. Each spectrum generally shows two well-defined peaks (except for the smallest diameters that more clearly shows a spectral band due to the transition regions). The two peaks correspond to the Rayleigh waves *R*_01_ and *TR*_21_ (coloured in blue and red, respectively). The *TR*_21_ peak is observed in all experimental spectra, in agreement with the theory, since there is no BSC expected for this mode. In contrast, the *R*_01_ is observed for small and large diameters—however, it is not observed (within the experimental noise limit) in the region around 1.1 μm—a clear evidence of the BSC effect. This is more readily observed in Fig. 4c, where the total scattered power in the *R*_01_ mode (*P*_01_) is normalized to the total scattered power in the *TR*_21_ mode (*P*_21_). Such normalization eliminates the uncertainty with respect to parameters that directly influence the absolute scattered power, such as slight differences in wire length, differences in the transition region optical attenuation, exact power at the wire input and the wire attenuation itself. The solid curve is the theoretical result, which is simply given by 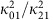. A remarkable agreement is observed and clearly the *R*_01_ mode intensity approaches the noise limit at 1.1 μm, confirming the BSC effect. To confirm that the observed peaks are due to the fundamental Rayleigh waves *R*_01_ and *TR*_21_, their central frequencies were measured and compared with the theoretical prediction (inset of Fig. 4c), with quite good agreement. This particular frequency versus diameter evolution arises from a trade-off between how fast the optical effective index drops and how fast the phase velocity of acoustic Rayleigh waves increases as diameter becomes smaller (see results in the Supplementary Fig. 4 and details in the Supplementary Note 3).

## Discussion

**Figure 5:**
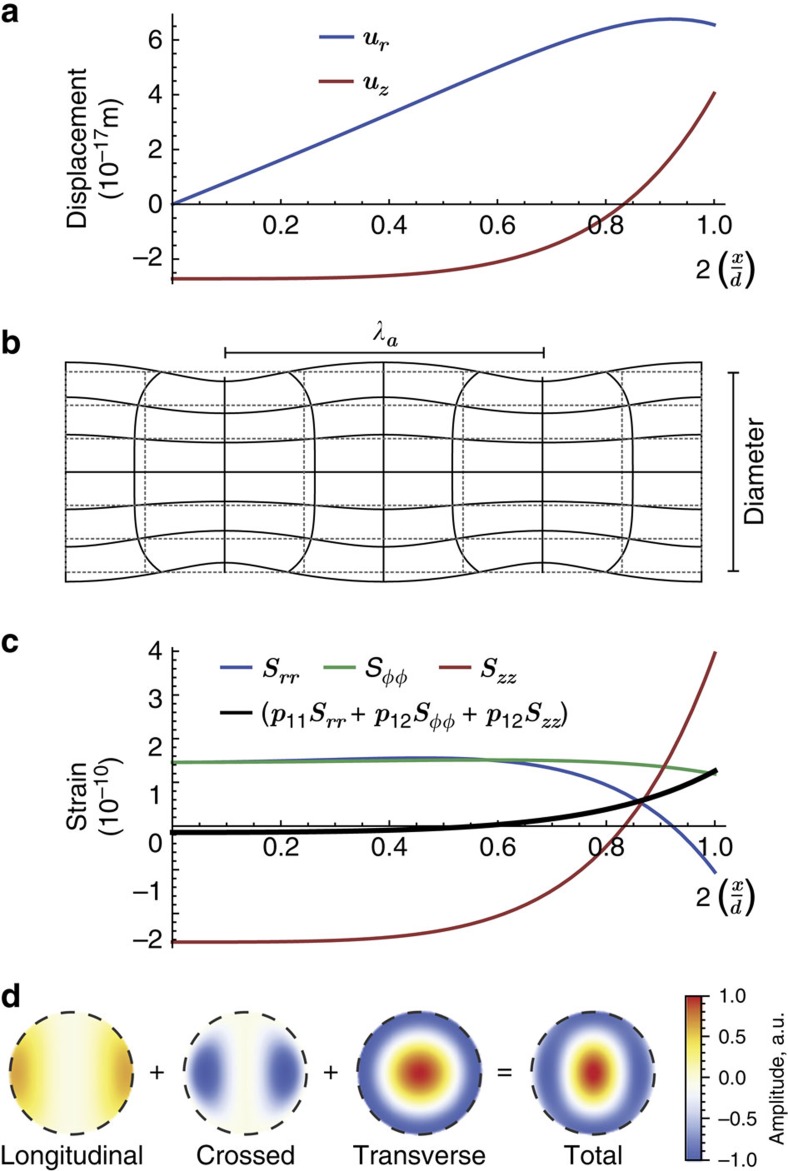
Physical understanding of the photo-elastic contribution. (**a**) Radial and axial displacement field profile for the fundamental *R*_01_ acoustic mode for a wire with *d*=1.1 μm diameter (absolute amplitude given by thermal energy normalization); (**b**) illustrative wire deformation (λ_a_ is the acoustic wavelength); (**c**) resulting strain profiles along with the total strain profile weighted by the photo-elastic coefficients; (**d**) each integrand term in the coupling coefficient overlap integral (equation 1) is shown separately (all in the same colour scale, in arbitrary units). The longitudinal term 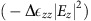 mostly cancels out the crossed term 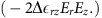. As a result, the total perturbation profile is mostly dominated by the transverse term 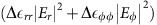.

To understand the cancellation effect in detail, it is essential to understand the acoustic profile and associated index perturbation profile. The fundamental *R*_01_ mode has no dependence on the azimuthal angle *φ*, and its displacement components are the axial 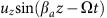 and the radial 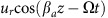. Figure 5a shows the transverse profile for each component and Fig. 5b illustrates the wire deformation under this particular mode (with amplitude arbitrarily large for better visualization). The maximum radial displacement, shown in Fig. 5a, is near the wire surface and therefore induces significant mb-effect. As mentioned before, the BSC effect shall occur only if the pe-effect causes a negative net dielectric perturbation to compensate the positive mb-effect perturbation. For the *R*_01_ mode, the only non-zero strain components are the diagonal terms *S*_*rr*_=∂_*r*_*u*_*r*_ (strain in the radial direction due to a radial displacement), *S*_*φφ*_=*u*_*r*_/*r* (strain in the azimuthal direction due to a radial displacement) and *S*_*zz*_=∂_*z*_*u*_*z*_ (strain in the axial direction due to axial displacement) and the cross-term 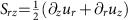. The strain profiles are shown in Fig. 5c. The only non-zero terms in the dielectric perturbation tensor are also the diagonal terms 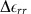, 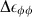 and 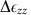, and the cross-term 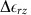. Explicitly, the diagonal term is 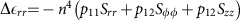. Similar expressions hold for 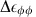, 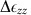 and 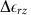 components. Note that the sign of the photo-elastic coefficients determines whether a positive (tensile) strain increases or reduces the dielectric constant. In silica (which is an isotropic medium), there are only two independent photo-elastic coefficients, *p*_11_ and *p*_12_, and they are both positives (except for the small crossed term 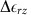 that depends on 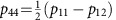, which is negative). Therefore, an expansion leads mostly to a reduction in the dielectric constant. More precisely, this means that 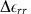 is negative if the sum of the strains (weighted by the photo-elastic coefficients) is positive. By examining the wire deformation profile in Fig. 5b, it becomes very clear which regions have positive or negative strain fields. In the centre of the wire, while it is compressed in the axial direction, it expands out radially (and thus azimuthally). This means that these strain fields counteract each other in the central region, leading to relatively smaller net contribution to the dielectric perturbation in the centre of the wire, as shown in the black solid curve in Fig. 5c. Note that this is completely counter-intuitive for those accustomed to pe-effect in conventional fibres, where axial strain dominates the effect. Near the wire surface, the strain competition picture changes radically. There is mostly simultaneous expansion in all (axial, azimuthal and radial) directions, and thus the strain fields contribute to a reduction on the dielectric constant (as required to achieve the BSC effect). One particular aspect for backward scattering (as opposed to forward scattering) is that *E*_*z*_—the longitudinal component of the electric field—changes sign for the backward wave. In cylindrical coordinates, integrand in equation 1 then becomes 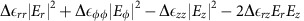. These terms are shown in the two-dimensional profile in Fig. 5d (normalized to unit). For this particular diameter, the last two terms (longitudinal and crossed-terms) mostly cancel out each other, and the net result is dominated by the transverse terms 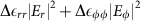. Moreover, the transverse optical field profile provides the right balance between the positive (central) and negative (edges) perturbation regions. As a result, for a nanowire with 1.1 μm diameter, the negative outer region dominates over the positive centre region by just the exact amount necessary to cancel the positive dielectric perturbation due to the mb-effect. This same dynamics explains why the pe-effect has a zero-point near 0.51 μm diameter (as previously shown in Fig. 2f), when the positive central region just balances out the negative outer region. Note that Fig. 4b shows the measured spectrum of a 0.51 μm diameter nanowire, the point where the Brillouin scattering is ‘totally' due to the mb-effect.

In conclusion, we have demonstrated experimentally the BSC effect in silica nanowires. Precise control of both optical and acoustic field profile turns out to create exactly opposing contributions due to pe- and mb-effects. In cylindrical geometry, we have demonstrated this effect for the fundamental axial–radial Rayleigh acoustic mode. A detailed understanding of the observed Brillouin spectrum and of the physical mechanism behind the cancellation effect is presented. We have also demonstrated that for 0.51 μm diameter, the pe-effect is zero (to first order) and the observed Brillouin scattering is produced ‘completely' by mb-effect only. Such rich interplay between pe- and mb-effects can be further explored by modal engineering and the use of materials with different photo-elastic coefficients and refractive indices to observe the effect of BSC in a variety of structures. Moreover, it can be used as a powerful tool to selectively control the photon–phonon interaction in photonic waveguides and cavities. From a broader perspective, our work solidifies the physical understanding of the two effects, and therefore, it supports not only the case when they act in opposite direction (leading to cancellation), but also when they act in the same direction (leading to an enhanced interaction)^17^,^18^,^19^,^20^,^23^. In this sense, the technological impact can be quite significant as both effects can be explored to design enhanced nano-photonic devices based on photon–phonon interaction such as narrow-band filters, acousto-optic modulators, microwave signal and frequency combs generation, Brillouin amplifiers and lasers, sensors and others.

## Methods

### Diameter characterization

Forward Brillouin scattering arises from an acoustic wave that oscillates transversally at the cut-off point, and therefore the observed frequency shift is inversely proportional to the wire diameter^15^,^40^,^43^. By measuring the forward-scattering frequency, we can accurately determine the wire diameter. A schematic of the setup is shown in Supplementary Fig. 1. In our experiment, the acoustic waves are excited using a pulsed-laser source (pulse duration of 25 ps, repetition rate of 1 MHz and wavelength at 1,570 nm) coupled with a probe continuous laser operating at 1,550 nm before entering the silica nanowire. At the output, the pump laser is filtered out and the probe inserted into a polarizer to convert polarization modulation into amplitude modulation. In this detection scheme, we observe polarization modulation arising from the fundamental torsional-radial acoustic mode (*TR*_21_). The modulated signal is then amplified with an optical pre-amplifier and detected in a high-speed photodiode. Finally, the signal is analysed in an electrical spectrum analyser and the peak frequency determined. An example of the detected spectrum (for a wire with 0.91 μm) is shown in Supplementary Fig. 2 and additional details are provided in the Supplementary Note 2.

### Backward Brillouin spectrum detection setup

The setup used to characterize the backward Brillouin spectrum is similar to the one described in ref. 2, and schematic is shown in Supplementary Fig. 3. A 1,550 nm narrow-linewidth diode laser (∼100 kHz linewidth) was amplified and launched in the silica nanowires. Light is launched into the wires using a circulator, to collect the backscattered Brillouin signal. Along with the frequency shifted Brillouin signal, a small linear component of the pump signal (non-frequency shifted) is also collected in the circulator. This is used as a reference for heterodyne detection. These two signals (reference and Brillouin signal) are amplified using a low-noise Erbium doped fibre pre-amplifier, detected in a high-speed PIN photodiode (>20 GHz bandwidth), amplified electrically in a low-noise radio-frequency pre-amplifier and dispersed in an electrical spectrum analyser. Additional details are provided in the Supplementary Note 2.

### Data availability

The data that support the findings of this study are available from the corresponding author on request.

## Additional information

**How to cite this article:** Florez, O. *et al*. Brillouin scattering self-cancellation. *Nat. Commun.* 7:11759 doi: 10.1038/ncomms11759 (2016).

## Supplementary Material

Supplementary Information Supplementary Figures 1-4, Supplementary Notes 1-3 and Supplementary References.
