# Supplementary material for: Brillouin scattering self-cancellation
Source: Nat Commun. 2016 Jun 10;7:11759. doi: 10.1038/ncomms11759 (PMC4906398; doi:10.1038/ncomms11759)
Supplement: Supplementary Information — Supplementary Figures 1-4, Supplementary Notes 1-3 and Supplementary References. [file ncomms11759-s1.pdf]

SUPPLEMENTARY FIGURES

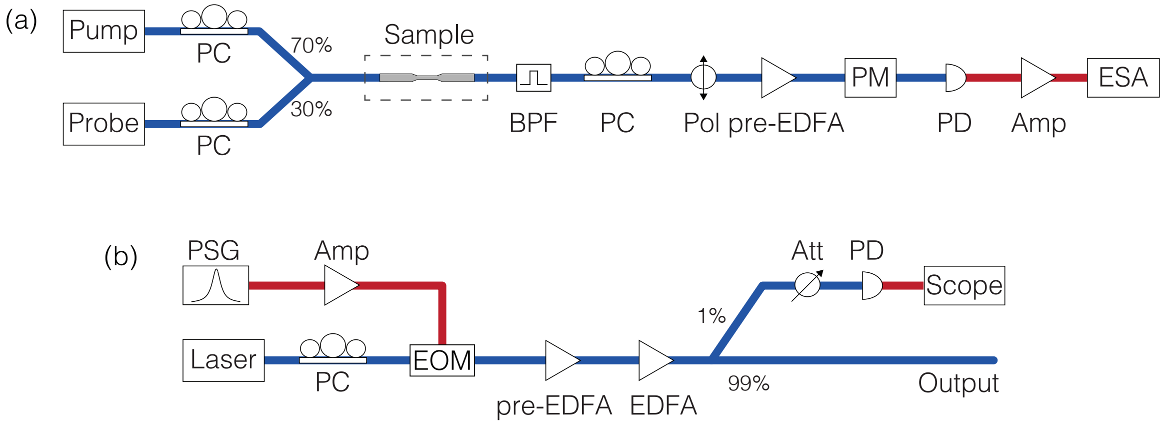

Supplementary Figure 1. Experimental setup used to characterize the forward Brillouin spectrum is shown in (a). The pulsed pump is generated using the setup in (b). PC: polarization controller; AMP: electrical amplifier; EOM: electro-optic modulator; EDFA (erbium-doped fiber amplifier); PM: in-line powermeter; BPF: band-pass optical filter; Pol: polarizer; Att: optical attenuator; PD: photodiode; and ESA: electrical spectrum analyzer.

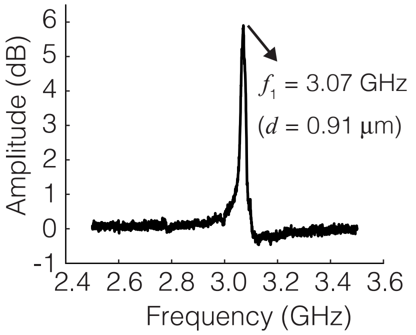

Supplementary Figure 2. Forward Brillouin spectrum arising from the fundamental TR_{21} acoustic mode. From the measured peak frequency (3.06 GHz), the wire diameter was determined to be 0.91 µm.

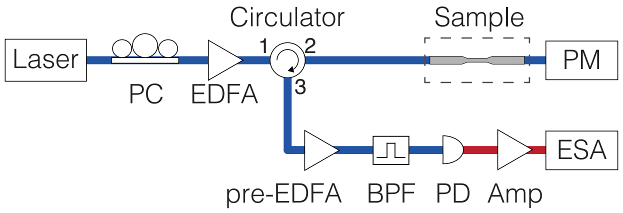

Supplementary Figure 3. Experimental setup used to characterize the Brillouin backscattering spectrum. PC: polarization controller; EDFA (Erbium-doped fiber amplifier); PM: powermeter; BPF: band-pass optical filter; PD: photodiode; AMP: electrical amplifier; and ESA: electrical spectrum analyzer.

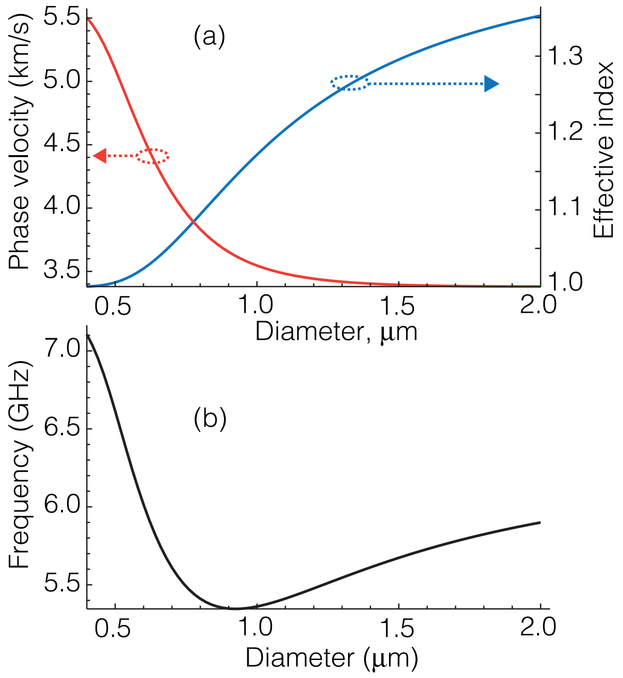

Supplementary Figure 4. (a) Optical mode effective index and acoustic mode phase velocity for the R_{01} mode as a function of wire diameter. In (b), the resultant Brillouin frequency shift is shown as a function of diameter.

SUPPLEMENTARY NOTE 1: NONLINEAR POLARIZATION

The macroscopic Maxwell equations in the time domain for a nonmagnetic medium with no free-charges are:

$$\begin{aligned} \nabla \cdot \mathbf{D} &= 0, \\ \nabla \cdot \mathbf{H} &= 0, \\ \nabla \times \mathbf{E} &= -\mu_0 \partial_t \mathbf{H}, \\ \nabla \times \mathbf{H} &= \partial_t \mathbf{D}. \end{aligned} \tag{1}$$

Here $\mathbf{D} = \varepsilon_0 \epsilon \mathbf{E} + \mathbf{P}_{\mathrm{NL}}$ is the electric displacement field, ε_0 is the vaccum permittivity, ϵ is the relative dielectric permittivity and μ_0 is the vacuum permeability. The nonlinear polarization $\mathbf{P}_{\mathrm{NL}}$ induced by an acoustic wave is written in the time domain as:

$$\mathbf{P}_{\mathrm{NL}}(t) = \varepsilon_0 \Delta\boldsymbol{\epsilon}(t) \cdot \mathbf{E}(t), \tag{2}$$

where $\Delta\boldsymbol{\epsilon}$ is the relative permittivity perturbation caused by the acoustic wave. For a harmonic perturbation, the relative permittivity perturbation can be written as:

$$\Delta\boldsymbol{\epsilon}(t) = \frac{1}{2} \Delta\boldsymbol{\epsilon}(x, y) e^{-i(\Omega t - \beta_a z)} + c.c., \tag{3}$$

where $\Delta\boldsymbol{\epsilon}(x, y)$, Ω and β_a are the perturbation transverse profile, angular frequency and propagation constant respectively. Since our interest is to investigate backward Brillouin scattering, we write the total field as the sum of a pump signal at ω_{p} and a backward propagating signal at ω_{s}. We restrict our development to the Stokes line so that $\omega_{\mathrm{s}} = \omega_{\mathrm{p}} - \Omega$ (generalization to the anti-Stokes line at $\omega_{as} = \omega_{\mathrm{p}} + \Omega$ is straightforward). We use the sub-index p to denote the pump field (propagating in the forward direction) and s to denote the scattered field propagating in the backward direction. We assume both fields as pure harmonics:

$$\begin{aligned} \mathbf{E}_{\mathrm{p}}(\mathbf{r}, t) &= \frac{1}{2} a_{\mathrm{p}}(z) \mathbf{E}_{\mathrm{p}}(x, y) e^{-i(\omega_{\mathrm{p}} t - \beta_{\mathrm{p}} z)} + c.c. \\ \mathbf{E}_{\mathrm{s}}(\mathbf{r}, t) &= \frac{1}{2} a_{\mathrm{s}}(z) \mathbf{E}_{\mathrm{s}}(x, y) e^{-i(\omega_{\mathrm{s}} t + \beta_{\mathrm{s}} z)} + c.c.. \end{aligned} \tag{4}$$

Note that the power carried by each signal is simply $P_{\mathrm{n}} = s_{\mathrm{n}} \frac{1}{2} |a_{\mathrm{n}}|^2 Re \left[\int \left(\mathbf{E}_{\mathrm{n}} \times \mathbf{H}_{\mathrm{n}}^* \right) \cdot \hat{\mathbf{z}} dA \right]$. From now on, $\mathbf{E}_{\mathrm{p,s}}$ denotes the transverse field profile $\mathbf{E}_{\mathrm{p,s}}(x, y)$. The factor $s_{\mathrm{p}} = +1$ for

the pump (co-propagating) and $s_{\mathrm{s}} = -1$ for the Stokes signal (back-propagating) are used so that the optical power is always positive. We can always normalize the eigenmodes so that $s_{\mathrm{n}} \frac{1}{2} Re \left[\int \left(\mathbf{E}_{\mathrm{n}} \times \mathbf{H}_{\mathrm{n}}^{*} \right) \cdot \hat{\mathbf{z}} dA \right] = 1$ W, and then at any position along the waveguide the power carried by the eigenmode n is simply $P_{\mathrm{n}} = |a_{\mathrm{n}}|^{2} \cdot 1$ W. We calculate the polarization generated by the pump field (i.e. neglecting the nonlinear polarization caused by the Stokes and anti-Stokes fields since in our experiments they are much weaker than the pump), obtaining:

$$\mathbf{P}_{\mathrm{NL}}(t) = \frac{\varepsilon_0 \Delta\epsilon}{4} a_{\mathrm{p}} \mathbf{E}_{\mathrm{p}}(x,y) e^{-i[\omega_{as} t - (\beta_{\mathrm{p}} + \beta_a) z]} + \frac{\varepsilon_0 \Delta\epsilon^{*}}{4} a_{\mathrm{p}} \mathbf{E}_{\mathrm{p}}(x,y) e^{-i[\omega_{\mathrm{s}} t - (\beta_{\mathrm{p}} - \beta_a) z]} + c.c.. \quad (5)$$

The first and the second terms are respectively the source terms for the anti-Stokes and Stokes scattered signals. Since we neglected the nonlinear polarization caused by the Stokes and anti-Stokes, there is no source term at the pump frequency ω_{p} (neither at high-order scattering such as $\omega_{\mathrm{p}} \pm 2\Omega$). This approximation implies a constant pump field amplitude along the waveguide, $a_{\mathrm{p}}(z) = a_{\mathrm{p}}$ (in other words, pump depletion is neglected).

Perturbation theory

The nonlinear polarization is treated as a perturbation term in Maxwell equations. We apply standard perturbation theory to calculate the evolution of the Brillouin backscattered signal amplitude $a_{\mathrm{s}}(z)$ along the waveguide. In the linear regime ($P_{\mathrm{NL}} = 0$), the macroscopic Maxwell equations for a harmonic field $Ee^{i\omega t}$ (specifically the curl equations in Supplementary Equation 1) can be written in terms of operators as:

$$A |\psi\rangle = -i\partial_z B |\psi\rangle , \quad (6)$$

where $|\psi\rangle = \begin{bmatrix} \mathbf{E} \\ \mathbf{H} \end{bmatrix}$, and the operators are defined as:

$$A = \begin{bmatrix} \omega\varepsilon_0\epsilon & -i\nabla_t \times \\ i\nabla_t \times & \omega\mu_0 \end{bmatrix}, \quad (7)$$

$$B = \begin{bmatrix} 0 & -\hat{\mathbf{z}}\times \\ \hat{\mathbf{z}}\times & 0 \end{bmatrix}. \quad (8)$$

For a waveguide, the solution to Supplementary Equation 6 is of the form:

$$|\psi_{\rm n}\rangle = \frac{1}{2} a_{\rm n} e^{i\beta_{\rm n} z} \, |\varphi_{\rm n}\rangle \, , \text{ with } \, |\varphi_{\rm n}\rangle = \begin{bmatrix} \mathbf{E}_{\rm n}(x,y) \\ \mathbf{H}_{\rm n}(x,y) \end{bmatrix} , \tag{9}$$

where $\mathbf{E}_{\boldsymbol{n}}$ and $\mathbf{H}_{\boldsymbol{n}}$ are the field profiles for a specific eigenmode, and $\beta_{\rm n}$ is the propagation constant (eigenvalue) at frequency ω. In the linear regime $a_{\rm n}$ is a constant. Substituting 9 into 6, we obtain a generalized eigenvalue problem:

$$A\,|\varphi_{\rm n}\rangle = \beta_{\rm n} B\,|\varphi_{\rm n}\rangle \, , \tag{10}$$

which for the back-propagating Stokes line becomes $A\,|\varphi_{\rm s}\rangle = -\beta_{\rm s} B\,|\varphi_{\rm s}\rangle$ (where the negative sign arises naturally from the $e^{-i\beta_{\rm s} z}$ dependence). The nonlinear polarization can be formally introduced as a perturbation term ΔA added to the operator A. For instance, from all the terms in the expression for the nonlinear polarization, the term oscillating at the Stokes field ($e^{-i\omega_{\rm s} t}$) is:

$$\mathbf{P}_{\rm NL}(t) = \frac{\varepsilon_0 \Delta\epsilon^*}{4} a_{\rm p} \mathbf{E}_{\rm p}(x,y)\, e^{-i[\omega_{\rm s} t - (\beta_{\rm p} - \beta_a) z]}, \tag{11}$$

In the presence of this term, Maxwell equations for the field oscillating as $e^{-i\omega_{\rm s} t}$ can again be cast in operator form as:

$$a_{\rm s} e^{-i\beta_{\rm s} z} A\,|\varphi_{\rm s}\rangle + a_{\rm p} e^{i(\beta_{\rm p} - \beta_a) z} \Delta A\,|\varphi_{\rm p}\rangle = -i\left(\partial_z a_{\rm s} - i\beta_{\rm s} a_{\rm s}\right) e^{-i\beta_{\rm s} z} B\,|\varphi_{\rm s}\rangle \, , \tag{12}$$

where the perturbation operator is:

$$\Delta A = \begin{bmatrix} \frac{\omega_{\rm s}\varepsilon_0}{2}\Delta\epsilon^* & 0 \\ 0 & 0 \end{bmatrix} . \tag{13}$$

Using the generalized eigenvalue equation (Supplementary Equation 10) for the Stokes signal, the first term on the left-hand side of Supplementary Equation 12 cancels with the second term on the right-hand side, which leads to:

$$\partial_z a_{\rm s} = -i\kappa a_{\rm p} e^{i\Delta\beta z}, \text{ with } \kappa = \frac{\langle\varphi_{\rm s}|\Delta A|\varphi_{\rm p}\rangle}{4} . \tag{14}$$

The phase mismatch is $\Delta\beta = \beta_{\rm p} + \beta_{\rm s} - \beta_a$ and κ is the coupling coefficient. In the definition of the coupling coefficient κ, we used the mode normalization

$$\langle\varphi_{\rm s}|B|\varphi_{\rm s}\rangle = 2\Re\left[\int \left(\mathbf{E}_{\rm n} \times \mathbf{H}_{\rm n}^*\right) \cdot \hat{\mathbf{z}} dA\right] = 4s_{\rm s}[\mathrm{W}] = -4[\mathrm{W}], \tag{15}$$

since $s_\mathrm{s} = -1$. Note that this normalization guarantees that the unit of κ is m^{-1}. The solution to Supplementary Equation 14 is:

$$a_\mathrm{s}(0) = -i\kappa a_\mathrm{p}\frac{1-e^{i\Delta\beta L}}{i\Delta\beta}, \tag{16}$$

$$P_\mathrm{s} = P_\mathrm{p}|\kappa|^2 L^2 \mathrm{sinc}^2\left(\frac{\Delta\beta L}{2}\right). \tag{17}$$

We have assumed that $a_\mathrm{s}(L) = 0$, where L is the waveguide length, and P_p is the pump power. In particular, for the case of perfect phase-matching $\Delta\beta = 0$, the total backscattered signal amplitude and power are given by:

$$a_\mathrm{s} = -ia_\mathrm{p}\kappa L, \tag{18}$$

$$P_\mathrm{s} = P_\mathrm{p}|\kappa|^2 L^2. \tag{19}$$

Although one might be drawn to conclude that the scattered power increases with the square of the waveguide length L, this is not correct. We show below that acoustic normalization (with $k_B T$ of energy per mode) results in a $L^{-1/2}$ dependence for the coupling coefficient, which combined with the L^2 factor in Supplementary Equation 19, yields a linear dependence of scattered power on the waveguide length.

The calculation of the inner product $\langle\varphi_\mathrm{s}|\Delta A|\varphi_\mathrm{p}\rangle$ in the coupling coefficient (Supplementary Equation 14) must be performed with care. For the photo-elastic effect, the perturbation $\Delta\epsilon$ caused by a mechanical strain is sufficiently small so that κ is simply

$$\kappa_\mathrm{pe} = \frac{\omega_\mathrm{s}\varepsilon_0}{8}\int \mathbf{E}_\mathrm{s}^*\cdot\Delta\boldsymbol{\epsilon}_\mathrm{pe}^*\cdot\mathbf{E}_\mathrm{p}dA, \tag{20}$$

where $\Delta\boldsymbol{\epsilon}_\mathrm{pe} = -n^4\mathbf{p}\cdot\mathbf{S}$. However, in the case of a moving boundary, $\Delta\boldsymbol{\epsilon}$ is simply $n_1^2 - n_2^2$, the difference in the relative permittivity between region 1 (wire core) and region 2 (air cladding). This perturbation is not small even for infinitesimal acoustic displacement and the field discontinuity must be taken into consideration. In this case, the correct expression for the coupling coefficient is [1]:

$$\kappa_\mathrm{mb} = \frac{\omega_\mathrm{s}\varepsilon_0}{8}\oint(\mathbf{u}\cdot\hat{\mathbf{n}})\left[\Delta\epsilon_\mathrm{mb}^*\mathbf{E}_{\mathrm{s},\parallel}^*\cdot\mathbf{E}_{\mathrm{p},\parallel} + \Delta\left(\epsilon_\mathrm{mb}^{-1}\right)^*\mathbf{E}_{\mathrm{s},\perp}^*\cdot\mathbf{E}_{\mathrm{p},\perp}\right]dl, \tag{21}$$

where $\Delta\epsilon_\mathrm{mb} = n_1^2 - n_2^2$ and $\Delta\left(\epsilon_{mb}^{-1}\right) = \left(n_2^{-2} - n_1^{-2}\right)n_2^4$ and the normal component of the electric field is evaluated in the region 2 (air cladding) to correctly take into account the field discontinuity. Note that $\mathbf{u}\cdot\hat{\mathbf{n}}$ is the normal component of the acoustic displacement calculated at the boundary, which in a cylindrical nanowire is simply the radial displacement

component at the boundary u_s. The integral is performed along the waveguide circular boundary. Supplementary Equations 20 and 21 were used to calculate the coefficients in Figures 2d-f. In these figures, κ was calculated for acoustic modes normalized to thermal energy (at 300 K), and the factor $L^{1/2}$ scales the normalization to any waveguide length (this is developed in detail in the next section). For example, Figure 2d gives the product $\kappa L^{1/2}$ for a wire with with 0.55 µm in diameter and so, for the R_{01} acoustic mode, $\kappa L^{1/2} = 4.5{\cdot}10^{-5}\ \mathrm{m}^{-1/2}$. Using $L = 0.08$ m, we then obtain $\kappa = 1.6{\cdot}10^{-4}\ \mathrm{m}^{-1}$. The total scattered power can be calculated using Supplementary Equation 19, and for a 1 W input pump power, the Stokes scattered power is approximately 170 pW.

Acoustic mode normalization and scattered light spectrum

Each acoustic mode carries a $k_B T$ of energy. Since the time average kinetic and potential energies are equal, we simply normalize the acoustic mode by making its average kinetic energy as $\mathcal{E}_k = \frac{1}{2}k_B T$. We write the acoustic field for a given mode identified with a sub-index l as:

$$\mathbf{u}_l\left(x,y,z,t\right) = \frac{1}{2}u_l \mathbf{U}_l\left(x,y\right)\ e^{i(\Omega_l t-\beta_l z)} + c.c., \tag{22}$$

where, u_l is the field amplitude (units of m) and $U_l\left(x,y\right)$ is the transverse mode profile (adimensional) normalized so that $\max\left|U_l\left(x,y\right)\right| = 1$, and β_l is the propagation constant of mode l evaluated at the acoustic frequency Ω_l. The average kinetic energy is then:

$$\mathcal{E}_k = \int \frac{1}{2}\rho\left\langle\left|\frac{\partial \mathbf{u}_l}{\partial t}\right|^2\right\rangle dV = \frac{1}{4}\rho\Omega_l^2|u_l|^2 L\int\left|U_l\left(x,y\right)\right|^2 dA \Rightarrow |u_l|^2 = \frac{4\mathcal{E}_k}{\rho\Omega_l^2 A_l L}, \tag{23}$$

where $\mathcal{E}_k = \frac{1}{2}k_B T$ and $A_l = \int\left|U_l\left(x,y\right)\right|^2 dA$. The last integral is performed over the waveguide cross-section area. We redefine the coupling coefficient so that the dependence on the acoustic amplitude u_l (determined by thermal energy) becomes explicit:

$$\kappa = k u_l. \tag{24}$$

In this way, k is fully determined by the modal profiles (optical and acoustic), and has unit of m^{-2}. Explicitly, for the moving boundary effect we have:

$$k_{\mathrm{mb}} = \frac{\omega_s \varepsilon_0}{8}\oint dl\left(\mathbf{U}_l{\cdot}\hat{\mathbf{n}}\right)\left[\left(n_1^2-n_2^2\right)\mathbf{E}^*_{s,\parallel}{\cdot}\mathbf{E}_{p,\parallel} + \left(n_1^2-n_2^2\right)\frac{n_2^2}{n_1^2}\mathbf{E}^*_{s,\perp}{\cdot}\mathbf{E}_{p,\perp}\right]dl. \tag{25}$$

Similarly, for the photo-elastic effect we have:

$$k_{\mathrm{pe}} = \frac{\omega_s \varepsilon_0}{8}\int \mathbf{E}^*_s{\cdot}\left(-n^4\mathbf{p}{\cdot}\boldsymbol{\mathcal{S}}\right){\cdot}\mathbf{E}_p dA, \tag{26}$$

where $\mathcal{S}_{ij}=\frac{1}{2}\left(\frac{\partial}{\partial x_j}U_{l,i}\ e^{-i\beta_l z}+\frac{\partial}{\partial x_i}U_{l,j}\ e^{-i\beta_l z}\right)$ is the strain created by the acoustic mode l divided by the thermal amplitude of the field u_l. With this definition, the total scattered power (assuming perfect phase-matching) becomes:

$$P_\mathrm{s}=P_\mathrm{p}|k_l|^2 L\frac{4\mathcal{E}_k}{\rho\Omega_l^2 A_l}=P_\mathrm{p}|k_l|^2 L\frac{2k_B T}{\rho\Omega_l^2 A_l}. \tag{27}$$

In deriving Supplementary Equation 27, we assumed that the acoustic field is harmonic and therefore the spectrum of the scattered light is single-frequency. In the presence of dissipation, the acoustic energy will be distributed over a Lorentzian spectrum. In order to calculate backscattered light spectrum, we simply write the acoustic energy spectrum density as

$$\mathbb{E}\left(\Omega\right)=\mathcal{E}_k\frac{\Gamma/\pi}{\left(\Omega-\Omega_l\right)^2+\Gamma^2}, \tag{28}$$

so that $\int_0^\infty \mathbb{E}\left(\Omega\right)d\Omega=\mathcal{E}_k$, and $\Gamma/2\pi$ is the Brillouin linewidth (in units of Hz). Therefore, the power spectrum density of scattered light becomes:

$$\mathbb{S}_{s,l}\left(\omega\right)=P_\mathrm{p}|k_l|^2 L\frac{2k_B T}{\rho\Omega_l^2 A_l}\frac{\Gamma/\pi}{\left(\omega-\omega_\mathrm{p}+\Omega_l\right)^2+\Gamma^2}. \tag{29}$$

For an instrument with frequency resolution Δf (in units of Hz), the measured power (in W) in a 1 Hz resolution will be:

$$\begin{aligned}P_\mathrm{s}[\text{W in bandwidth }\Delta f]&=\mathbb{S}_{s,l}\left(\omega\right)\Delta\omega=\mathbb{S}_{s,l}\left(\omega\right)2\pi\Delta f\\&=P_\mathrm{p}|k_l|^2 L\frac{2k_B T}{\rho\Omega_l^2 A_l}\frac{2\Gamma\Delta f}{\left(\omega-\omega_\mathrm{p}+\Omega_l\right)^2+\Gamma^2}.\end{aligned} \tag{30}$$

Finally, the scattered power per unit of frequency (W/Hz) normalized by the pump power and the waveguide length is:

$$\frac{\mathbb{S}_\mathrm{s}}{P_\mathrm{p}L}=|k_l|^2\frac{2k_B T}{\rho\Omega_l^2 A_l}\frac{2\Gamma}{\left(\omega-\omega_\mathrm{p}+\Omega_l\right)^2+\Gamma^2}. \tag{31}$$

This result is used to generate the theoretical Brillouin backscattering spectrum in Figures 3a, 3b and 4a.

SUPPLEMENTARY NOTE 2: EXPERIMENTAL SETUP

Diameter characterization

Forward Brillouin scattering arises from an acoustic wave that oscillates transversally at the cut-off point ($\beta_a = 0$). Therefore, the observed Brillouin frequency shift is exactly the acoustic cut-off frequency. For a cylindrical rod, the cut-off frequency is inversely proportional to the wire diameter [2–4], and can be obtained by applying the free-surface boundary condition. For example, for the axially asymmetric torsional-radial (TR_{2m}) mode, $f_m = 2y_m v_T/\pi d$, where y_m are the numerical solution of the transcendental equation:

$$\begin{aligned} &J_1(y)\left(8\left(\alpha^2-1\right)\left(y^2-6\right)J_1(y\alpha)+4\alpha y\left(y^2-6\right)J_0(y\alpha)\right)+ \\ &J_0(y)\left(2y\left(\alpha^2\left(y^2+12\right)+y^2-24\right)J_1(y\alpha)-\alpha y^2\left(y^2-24\right)J_0(y\alpha)\right)=0 \end{aligned} \tag{32}$$

Note that this equation depends only on the parameter $\alpha = v_T/v_L$, the ration between the shear and longitudinal bulk acoustic velocities, which for silica is approximately 0.63. The numerical solution for the fundamental TR_{21} mode is $y_1 = 2.34$. Therefore, by measuring the forward Brillouin scattering frequency shift for the TR_{21} mode, we can accurately determine the wire diameter by $d = 2y_1 v_T/\pi\, f_1$.

The experimental setup used to characterize the forward Brillouin spectrum on our nanowires is based on a pump-and-probe technique, and is shown in Supplementary Figure 1. The fundamental TR_{21} acoustic mode is excited using a pulsed laser source (pulse duration of 25 ps, repetition rate of 1 MHz and wavelength at 1570 nm). A probe continuous laser operating at 1550 nm is combined with the pump signal and launched into the silica nanowire. At the output, the pump laser is filtered out and the probe inserted into a polarizer in order to convert polarization modulation into amplitude modulation. The modulated signal is then amplified with an optical pre-amplifier and detected in a high-speed photodiode. Finally, the signal is analyzed in an electrical spectrum analyzer, and the peak frequency determined. The pulsed pump signal was generated using an Pulse Signal Generator and an Electro-Optical amplitude modulator. After modulation, the signal is pre-amplified using an pre-EDFA and then injected into a high-power EDFA (this is simply to saturate the high-power amplifier and reduce spontaneous emission). A small fraction ($\sim$)1% of the pump power is detected using a photodiode and monitored in a oscilloscope.

Supplementary Figure 2 shows an example of the forward Brillouin spectrum measured. The peak observed is due to the fundamental TR_{21} mode and the measured frequency is 3.07 GHz, from which the wire diameter was determined to be 0.91 µm. All samples were characterized using this methods.

Brillouin backscattering experimental setup

The setup used to characterize the backward Brillouin spectrum is similar to the one described in [5], and is shown in Supplementary Figure 3. A 1550 nm narrow-linewidth diode laser (∼100 kHz linewidth) was amplified with an EDFA (with 27 dBm output power) and launched into the silica nanowires using a circulator. The backscattered Brillouin signal is collected on port 3 of the circulator. Along with the frequency shifted Brillouin signal, a small linear reflection of the pump signal (non-frequency shifted) is used as a reference for heterodyne detection. These two signals (reference and Brillouin signal) are amplified using a low-noise Erbium doped fiber pre-amplifier, detected in a high-speed PIN photodiode ($>$ 20 GHz bandwidth), amplified electrically in a low noise radio-frequency pre-amplifier and dispersed in an electrical spectrum analyzer.

SUPPLEMENTARY NOTE 3: BRILLOUIN FREQUENCY SHIFT *vs.* WIRE DIAMETER

The evolution of the Brillouin frequency with diameter shown in Figure 4c can be understood by evaluating both optical and acoustic dispersion relations. The phase-matched acoustic frequency is $f_{ac} = 2n_{eff}v_{ac}/\lambda$, where λ is the optical wavelength, n_{eff} is the optical mode effective index and v_{ac} is the phase velocity for a given acoustic mode. Therefore, the Brillouin frequency behavior depends on how n_{eff} and v_{ac}vary with the nanowire diameter, as shown in Supplementary Figure 4a. The effective index decreases monotonically as the diameter is reduced due to increasing diffraction that spreads the optical energy out into the air cladding. Thus the effective index contributes to reduce the acoustic frequency f_{ac} as the diameter is reduced. On the other hand, the acoustic phase velocity v_{ac} always increases as the diameter is reduced (from the lower limit referred to as Rayleigh velocity v_R to its maximum bulk longitudinal value v_L), and thus its contribution is to increase the acoustic frequency f_{ac}. The resultant frequency is shown in Supplementary Figure 4b. Note that f_{ac} first decreases before it increases again when the diameter is reduced (from right to left in Supplementary Figure 4b). This behavior has its origin in the fact that that diffraction of the optical mode into the cladding occurs at a wire diameter comparable to the optical wavelength while the acoustic phase velocity is only affected when the wire diameter is comparable to the acoustic wavelength. Since the optical wavelength is twice the acoustic wavelength (as required by the phase-matching condition), it means that the reduction of n_{eff} impacts the Brillouin frequency f_{ac} before v_{ac} does. This trade-off between how fast the optical effective index drops and how fast the phase velocity of acoustic Rayleigh waves increases as diameter becomes smaller determines the behavior of the acoustic frequency observed in Figures 4c.

SUPPLEMENTARY REFERENCES

1. Johnson, S. G. *et al.* Perturbation theory for Maxwell's equations with shifting material boundaries. *Phys. Rev. E* **65,** 066611 (6 June 2002).

2. Shelby, R. M., Levenson, M. D. & Bayer, P. W. Guided Acoustic-Wave Brillouin-Scattering. *Physical Review B* **31,** 5244–5252 (1985).

3. Kang, M. S., Brenn, A., Wiederhecker, G. S. & Russell, P. S. Optical excitation and characterization of gigahertz acoustic resonances in optical fiber tapers. *Applied Physics Letters* **93,** 131110 (2008).

4. Dainese, P. *et al.* Raman-like light scattering from acoustic phonons in photonic crystal fiber. *Optics Express* **14,** 4141–4150 (May 2006).

5. Dainese, P. *et al.* Stimulated Brillouin scattering from multi-GHz-guided acoustic phonons in nanostructured photonic crystal fibres. *Nature Physics* **2,** 388–392 (June 2006).
